# Laterality and Breed Distribution of Cryptorchidism in 251 Dogs: A Retrospective Clinical Study

**DOI:** 10.3390/vetsci13050478

**Published:** 2026-05-15

**Authors:** Rafalska Agata, Domosławska Anna

**Affiliations:** Department of Animal Reproduction with Clinic, University of Warmia and Mazury in Olsztyn, 10-719 Olsztyn, Poland; anna.domoslawska@gmail.com

**Keywords:** dog, cryptorchidism, testicular descent, orchidectomy, diagnosis

## Abstract

Cryptorchidism is one of the most frequently diagnosed developmental disorders of the male canine reproductive system, defined as the failure of one or both testes to descend into the scrotum. Physiologically, testicular descent is typically completed by six to eight weeks of age, although some authors extend this period to sixteen weeks. Failure of testicular descent beyond this timeframe is considered pathological. The condition has multiple causes and affects between 1% and 10% of the canine population. Genetics is the most significant factor, indicating the hereditary basis of cryptorchidism. In addition, increasing attention has been directed toward the potential impact of environmental and epigenetic factors on the incidence of cryptorchidism, suggesting that the condition may result from complex interactions between genetic predisposition and external influences. The effect of hormones (such as INSL3 and testosterone), mechanical factors (including narrowing of the inguinal canal, abnormalities of the gubernaculum, and shortening of the spermatic cord), and environmental factors (for example, exposure to external estrogens and maternal stress during pregnancy) all contribute to the development of this disorder. Recent results have emphasized the role of the orexin system, particularly the OX2R receptor, in regulating endocrine and reproductive functions in cryptorchid testes. Computed tomography is increasingly utilized in complex cases due to its high precision in localizing retained testes. Clinically, cryptorchidism may present unilaterally or bilaterally. Unilateral cryptorchidism may preserve partial fertility, whereas bilateral cryptorchidism results in complete infertility. Undescended testes may be located in the abdominal cavity or inguinal canal. Major complications include an increased risk of testicular cancer (Sertoli cell tumors and seminomas) and endocrine disorders leading to feminization. Diagnosis is based on clinical examination and imaging modalities such as ultrasound. Orchiectomy, involving the removal of both the retained and normally descended testicles, is thought to be the gold standard for treatment. This method helps avoid complications and the transmission of the defect to offspring. According to Fédération Cynologique Internationale (FCI) standards, affected individuals should not be used for breeding or shows. Early detection, surgical intervention, and consistent exclusion from breeding programs are the primary strategies for reducing the incidence of this disorder in the canine population.

## Simple Summary

This retrospective study evaluates the distribution and laterality of cryptorchidism in 251 male dogs presented to a veterinary clinic in Poland between 2015 and 2025. Cryptorchidism, defined as the failure of one or both testes to descend into the scrotum, represents a common developmental disorder of the canine reproductive system with a multifactorial etiology, including genetic, hormonal, mechanical, and environmental influences. The findings demonstrate a marked predominance of unilateral cryptorchidism (94.8%), with bilateral cases accounting for only 5.2% of the study population. A strong asymmetry in laterality was observed, with right-sided cryptorchidism (81.3%) occurring substantially more frequently than left-sided cases (13.5%). The condition was identified across a broad range of breeds, with the highest frequencies noted in Golden Retrievers, Yorkshire Terriers, Labrador Retrievers, and mixed-breed dogs. These results confirm that cryptorchidism is not restricted to small breeds and may affect dogs of various sizes. From a clinical perspective, cryptorchidism is associated with an increased risk of testicular neoplasia, endocrine disorders, and impaired fertility. Orchiectomy remains the treatment of choice, and exclusion of affected individuals from breeding programs is strongly recommended due to the hereditary nature of the disorder. Overall, this study provides valuable epidemiological insight into breed distribution and laterality patterns of cryptorchidism in dogs.

## 1. Introduction

Cryptorchidism is the most prevalent developmental disorder of the male canine reproductive system. Testicular descent is a physiological process in which one or both testicles migrate to the scrotum, typically completed between 6 and 8 weeks of age. However, some authors extend this period to 16 weeks [1]. Failure of testicular descent beyond this period is considered pathological and may result in significant clinical and reproductive consequences. The clinical importance of cryptorchidism lies in the fact that it is associated with an increased risk of testicular cancer (Sertoli cell tumors, seminomas, and less commonly Leydig cell tumors) [2]. Testicles retained in the abdominal cavity or inguinal canal are also susceptible to testicular torsion, which requires immediate surgical intervention [3]. Bilateral cryptorchidism leads to infertility due to impaired spermatogenesis from prolonged exposure to elevated intra-abdominal temperatures [4,5]. Endocrine disorders additionally influence the clinical effects of cryptorchidism. Affected dogs, particularly those with Sertoli cell tumors, may develop male feminization syndrome, gynecomastia, symmetrical alopecia, decreased libido, and, in advanced cases, bone marrow suppression resulting in life-threatening aplastic anemia [2,6]. Ultrasonography plays an important role in the diagnostic work-up of suspected cryptorchidism [7]. Abdominal and inguinal ultrasound examination enables non-invasive localization of retained testes, particularly when they are not palpable on clinical examination [8,9]. The technique is especially valuable in differentiating intra-abdominal from inguinal retention and in identifying hypoplastic, neoplastic, or torsed testes [8]. Ultrasonography may also assist in preoperative planning by determining the exact position, size, echogenicity, and vascularization of the retained gonad [9]. In cases of suspected neoplastic transformation, Doppler evaluation can provide additional information regarding blood flow patterns, supporting early detection of pathological changes [2]. In cases where ultrasonography does not provide conclusive results or when complications such as neoplasia, spermatic cord torsion, or atypical testicular location are suspected, advanced imaging techniques may be indicated. Although ultrasonography may not always detect very small or atrophic testes, it remains a safe, widely available, and highly useful first-line diagnostic tool in clinical practice [7,10]. Computed tomography facilitates precise localization of the retained testis and assessment of its anatomical relationships, thereby supporting accurate preoperative planning [11]. The gold standard for this procedure is to perform an orchidectomy after first locating the testicles in the abdominal cavity or groin. Beyond clinical implications, cryptorchidism is a significant breeding and population-health concern. The disorder is hereditary, and affected dogs should be excluded from breeding programs to prevent transmission of genetic predisposition [12,13]. Fédération Cynologique Internationale (FCI) and national kennel organizations, such as the Polish Kennel Club, classify the absence of one or both scrotal testes as a disqualifying fault. The prevalence of cryptorchidism in dogs ranges from 1% to 10%, with considerable variation among breeds [13,14]. While most commonly observed in small and miniature breeds, including Yorkshire Terriers, Miniature Poodles, Chihuahuas, Pomeranians, and Dachshunds, cryptorchidism also occurs in large and giant breeds, such as German Shepherd Dogs and Great Danes, indicating it is not limited by body size [3,13]. Despite extensive research into its etiology and pathogenesis—including genetic, hormonal, mechanical, and environmental factors—population-based clinical data on breed distribution and laterality remain limited [15,16]. Recent molecular studies have identified altered expression of orexin receptors (OX2R) in cryptorchid testes, suggesting additional pathophysiological mechanisms that require further investigation [17,18]. The hormone INSL3 is a critical regulator of testicular descent in dogs, primarily through its role in promoting the development and differentiation of the gubernaculum, which facilitates the translocation of the testes into the scrotum. Alterations in the expression or signaling of this hormone are associated with impaired testicular descent and may contribute to the pathogenesis of cryptorchidism [19,20,21]. However, the clinical relevance of these findings at the population level remains unclear. The present study aims to conduct a retrospective analysis of dogs diagnosed with cryptorchidism in clinical practice, with particular focus on breed distribution and laterality of retained testes. By providing population-based data from a representative cohort, this study seeks to enhance understanding of the epidemiology of cryptorchidism in dogs and to inform clinical decision-making and responsible breeding strategies. The statistics presented in this paper primarily serve to highlight the fact that the problem of cryptorchidism persists despite advances in breeding and increased awareness among breeders.

## 2. Materials and Methods

### 2.1. Study Design

This study was a retrospective observational analysis utilizing veterinary clinical records from male dogs diagnosed with cryptorchidism at the Department of Animal Reproduction with Clinic, Faculty of Veterinary Medicine, University of Warmia and Mazury in Olsztyn, Poland. Data collection began in 2015 and ended in 2025. Cases identified through routine diagnostic examinations were included, with no experimental manipulation or prospective intervention. Inclusion criteria comprised male sex, clinical diagnosis of cryptorchidism established during general examination, absence of one or both testes from the scrotum beyond the physiological age for testicular descent, and complete clinical data sufficient for reliable assessment of breed and laterality. Both bilateral and unilateral forms of cryptorchidism were included, regardless of whether the retained testis was located in the inguinal canal or abdominal cavity. Cases of delayed physiological testicular descent in young puppies were excluded to prevent misclassification of non-pathological conditions. Additional exclusion criteria included a history of prior orchiectomy or castration, incomplete or ambiguous clinical documentation, congenital absence of one or both testes (monorchism or anorchism), and cases where the absence of scrotal testes could not be definitively distinguished from testicular ectopia. Due to the retrospective nature of the study, standardized diagnostic and therapeutic protocols could not be implemented; however, all diagnoses were based on accepted clinical criteria. No additional diagnostic tests or treatments were performed on any animals in this study. All diagnostic and surgical procedures documented in the clinical records were conducted solely for therapeutic and clinical purposes, unrelated to the research objectives.

### 2.2. Diagnostic Procedures

Diagnosis of cryptorchidism was established using a standard clinical approach consistent with veterinary practice. Each dog underwent a comprehensive physical examination, including a detailed inspection and palpation of the scrotum and inguinal region to assess the presence, size, and consistency of the testicles. When a retained testicle could not be identified by palpation, imaging diagnostics were used to locate the undescended gonad. Ultrasonography was the imaging method used to evaluate gonadal morphology and detect testicles retained in the inguinal canal or abdominal cavity. The examination was performed in the supine or standing position using a Mindray Bio-Medical 2 device (Mindray Bio-Medical Electonics Co., Ltd., Shenzhen, China) with a 7.5 MHz convex probe. This technique was also used to identify pathological changes in echogenicity suggesting neoplastic transformation. All ultrasound imaging procedures were performed for diagnostic or therapeutic purposes only, not as part of experimental studies.

### 2.3. Classification of Cryptorchidism

Cryptorchidism was determined based on clinical and imaging findings. Cases were classified as right-sided, left-sided, or bilateral cryptorchidism based on laterality. This classification was applied uniformly throughout the entire study population. For bilateral cryptorchidism, both testes were analyzed and assessed separately; however, laterality classification was determined by the presence of one or both undescended testes.

### 2.4. Data Collection

Clinical records were systematically reviewed, and data were extracted for each included dog. Variables collected included breed, number of retained testes, and laterality. Breed information was recorded as documented in the clinical records, with dogs of unknown or mixed ancestry grouped and analyzed separately. Data extraction followed standardized procedures to minimize discrepancies and reduce the risk of misclassification. Cases with incongruities or ambiguities in the clinical records were excluded from analysis based on the predetermined exclusion criteria.

### 2.5. Outcome Measures

The primary outcome measures were the distribution of cryptorchidism by laterality (right-sided, left-sided, bilateral) and by breed. Secondary outcome measures included the relative frequency of unilateral versus bilateral cryptorchidism. These outcomes were selected to characterize the epidemiological features of cryptorchidism in the studied canine population and to facilitate comparison with published data.

### 2.6. Statistical Analysis

Descriptive statistical methods were employed for data analysis. Categorical variables are presented as absolute numbers and percentages. As the study was retrospective and descriptive, no predefined hypotheses for inferential testing were formulated, and no statistical comparisons or multivariable analyses were performed. The analyses were designed to characterize the distribution of cryptorchidism rather than to determine causality.

### 2.7. Ethical Statement

As this study was based solely on a retrospective analysis of veterinary clinical records, ethical review and approval were waived. No experimental procedures, prospective interventions, or additional diagnostic tests were carried out for research purposes. All diagnostic and surgical procedures documented in the clinical records were performed strictly for therapeutic and diagnostic purposes, in accordance with accepted veterinary practice standards.

## 3. Results

### 3.1. Study Population

This retrospective analysis included 251 male dogs diagnosed with cryptorchidism between the ages of 8 months and 3 years. The mean age was 1.68 years. The study population comprised dogs of various breeds, including mixed breeds, all of whom were patients at the Department of Animal Reproduction with Clinic, Faculty of Veterinary Medicine, University of Warmia and Mazury in Olsztyn, Poland. Both unilateral and bilateral forms of cryptorchidism were identified within the cohort (Table 1).

### 3.2. Overall Distribution of Cryptorchidism

Unilateral cryptorchidism was the most prevalent form, diagnosed in 238 dogs (94.8%). Right-sided cryptorchidism occurred in 204 dogs (81.3%), while left-sided cryptorchidism was identified in 34 dogs (13.5%). Bilateral cryptorchidism was observed in only 13 dogs (5.2%) (Table 1).

### 3.3. Breed Distribution and Laterality

Marked differences were observed in the frequency and laterality of cryptorchidism among breeds. The highest proportions of affected dogs were found in Golden Retrievers (*n* = 41), followed by mixed-breed dogs (*n* = 29), Yorkshire Terriers (*n* = 27), Labrador Retrievers (*n* = 17), and smooth-haired Dachshunds (*n* = 14). Right-sided cryptorchidism predominated in most breeds. In several breeds, including the Chinese Crested Dog, Miniature Schnauzer, Longhaired Chihuahua, Miniature Poodle, and Alaskan Malamute, only right-sided cryptorchidism was observed. Greater variability in laterality was observed in Pomeranians and long-haired German Shepherd Dogs, with left-sided and bilateral cryptorchidism also occurring. Bilateral cryptorchidism was rare and recorded in only a few breeds, such as the Nova Scotia Duck Tolling Retriever and the Shorthaired Chihuahua. Additional breed-specific distribution and laterality data are presented in Table 1.

### 3.4. Summary of Laterality Patterns

Analysis of laterality patterns revealed a pronounced predominance of right-sided cryptorchidism. Among the 251 dogs evaluated, 204 (81.3%) had right-sided cryptorchidism, 34 (13.5%) had left-sided cryptorchidism, and 13 (5.2%) exhibited bilateral cryptorchidism. Unilateral cryptorchidism accounted for the majority of cases (238 dogs, 94.8%), with right-sided retention occurring approximately six times more frequently than left-sided retention. Most breeds demonstrated a predominance of right-sided cryptorchidism. In certain breeds, such as Chinese Crested Dogs, Miniature Schnauzers, Longhaired Chihuahuas, Miniature Poodles, Alaskan Malamutes, Border Collies, and Whippets, only right-sided cryptorchidism was observed. Left-sided and bilateral cryptorchidism were limited to a few breeds and occurred infrequently. Bilateral cryptorchidism was detected in Golden Retrievers, Pomeranians, long-haired German Shepherd Dogs, Nova Scotia Duck Tolling Retrievers, and Shorthaired Chihuahuas, with no breed exhibiting more than a single-digit number of cases. Overall, right-sided cryptorchidism was the most common laterality pattern, while bilateral and left-sided cases were considerably less frequent.

## 4. Discussion

This retrospective study provides population-based clinical data on cryptorchidism in dogs, focusing on breed distribution and the laterality of retained testes. The findings demonstrate that unilateral cryptorchidism is the predominant clinical presentation, with a marked predominance of right-sided testicular retention. Bilateral cryptorchidism was infrequent, accounting for only a small proportion of cases, consistent with previous epidemiological reports [4,22]. The predominance of right-sided cryptorchidism aligns with earlier studies describing asymmetry in testicular descent [1,9,23]. Developmental differences between the right and left testes, such as the later descent of the right testis during embryonic and early postnatal development, may increase susceptibility to disruptions in hormonal signaling, gubernacular development, or mechanical migration through the inguinal canal [12]. Although embryological mechanisms were not directly investigated, the consistent predominance of right-sided cryptorchidism across breeds supports the role of biological asymmetry in testicular descent. Notably, considerable breed-related variability in the occurrence and laterality of cryptorchidism was observed. Affected dogs represented a wide range of breeds, including both small and large breeds. Higher case numbers were recorded among Golden Retrievers, Yorkshire Terriers, Labrador Retrievers, and mixed-breed dogs. While previous studies have emphasized increased risk in small and miniature breeds [13,14], the presence of cryptorchidism in large and giant breeds in this study confirms that the disorder is not limited to small and miniature breeds [23,24]. These results support the hypothesis of a genetic predisposition to cryptorchidism, which may be independent of body size and instead reflect breed-specific developmental characteristics [13,25]. It should also be noted that this may depend on the relative prevalence and popularity of specific breeds in a given region. The fact that in some breeds this affects exclusively or almost exclusively the right testicle suggests breed-dependent patterns of testicular migration, as confirmed by previous clinical and anatomical studies [23]. However, the small number of cases in some breeds limits definitive conclusions regarding breed-specific risk. Bilateral cryptorchidism was rare and did not predominate in any breed, suggesting that complete failure of testicular descent may require more severe developmental disruption or multifactorial etiological influences [1,26]. Clinically, these findings underscore the importance of early diagnosis, even in dogs without overt abnormalities. Dogs with unilateral cryptorchidism may retain normal libido and partial fertility, potentially delaying recognition, especially in breeding animals [12]. Nevertheless, retained testes are associated with a substantially increased risk of neoplastic transformation and endocrine disorders, regardless of laterality [2,8,22]. Therefore, current recommendations for surgical removal of both retained and scrotal testes remain justified [22,27,28,29]. The breeding implications are significant; excluding affected dogs from breeding programs is important to prevent further dissemination of the genetic predisposition [13,14]. However, as is widely known, genetic affinity and inbreeding can result in cryptorchidism and other defects. Not all owners were interested in registering their males as future stud dogs (they did not know their dogs’ pedigrees) or they were mixed breeds, so only in some cases could a genetic problem related to too close a relationship be suspected. It was impossible to compile appropriate statistics in these cases. The widespread occurrence of cryptorchidism across multiple breeds highlights the need for ongoing breeder education, strict adherence to cynological regulations, and responsible reproductive selection as key components of disease prevention [25]. Several limitations should be acknowledged. The retrospective design limited analysis to available clinical parameters and precluded assessment of hormonal profiles, environmental influences, or long-term outcomes [15,30]. The study population consisted exclusively of dogs presented for veterinary evaluation, which may introduce selection bias and limit generalizability. Despite these limitations, the large sample size and broad breed representation provide meaningful insight into the epidemiology of cryptorchidism in dogs. Overall, the study confirms a pronounced predominance of right-sided unilateral cryptorchidism and substantial breed-related variability. These results contribute valuable epidemiological data and support current clinical and breeding management guidelines. Future prospective studies incorporating genetic, hormonal, and environmental factors are needed to elucidate further mechanisms underlying breed susceptibility and laterality patterns in cryptorchid dogs [4,23,25].

## 5. Conclusions

This retrospective study presents population-based data on the distribution and laterality of cryptorchidism in dogs. The results indicate that cryptorchidism is most often unilateral, with a marked right-sided predominance. Bilateral cryptorchidism was rare, occurring in only a small number of cases. Significant variation in the occurrence and laterality of cryptorchidism among breeds was observed, supporting the existence of breed-specific predispositions and a genetic basis for the disorder. Excluding affected dogs from breeding programs is essential to reduce the transmission of genetic predispositions. Despite the extensive knowledge already gathered about cryptorchidism in male dogs, research on its occurrence is still being conducted, and statistics show that such research is still important.

## Figures and Tables

**Table 1:** Breed distribution and laterality of cryptorchidism in 251 dogs.

Breed	Total (*n*)	Right Testicle (*n*)	Left Testicle (*n*)	Bilateral (*n*)
Golden retriever	41	34	2	5
Dachshund smooth	14	10	4	
Cairn terrier	1	1		
Yorkshire terrier	27	20	5	2
Chinese crested dog	12	12		
Alaskan malamute	5	5		
Miniature schnauzer	7	7		
Nova Scotia Duck Tolling Retriever	1			1
Pug	8	7	1	
Chihuahua longcoat	13	13		
Pomeranian	12	7	3	2
Dachshund rough	2	2		
Doberman	2	2		
Poodle miniature	4	4		
Chihuahua shorthaired	9	6	2	1
Australian Shepherd	2	2		
German Shepherd longhaired	11	7	3	1
Papillon	2	2		
Great Dane	7	5	2	
Border collie	2	2		
Cane Corso	1	1		
Cavalier KCS	6	4	2	
Maltese	2	2		
French bulldog	7	5	2	
Whippet	2	2		
Rottweiler	1	1		
Labrador retriever	17	13	4	
White Swiss Shepherd Dog	2	2		
Rhodesian ridgeback	2	2		
mix	29	24	4	1
Total	251	204	34	13

## Data Availability

All the data are presented within the paper.
